# Prenatal Stress Down-Regulates Reelin Expression by Methylation of Its Promoter and Induces Adult Behavioral Impairments in Rats

**DOI:** 10.1371/journal.pone.0117680

**Published:** 2015-02-13

**Authors:** Ismael Palacios-García, Ariel Lara-Vásquez, Juan F. Montiel, Gabriela F. Díaz-Véliz, Hugo Sepúlveda, Elías Utreras, Martín Montecino, Christian González-Billault, Francisco Aboitiz

**Affiliations:** 1 Departamento de Psiquiatría, Escuela de Medicina, and Centro Interdisciplinario de Neurociencia, Pontificia Universidad Católica de Chile, Santiago, Chile; 2 Centro de Investigación Biomédica, Facultad de Medicina, Universidad Diego Portales, Santiago, Chile; 3 Programa de Farmacología Molecular y Clínica, Instituto de Ciencias Biomédicas, Universidad de Chile, Santiago, Chile; 4 Laboratory of Cell and Neuronal Dynamics (Cenedyn), Department of Biology, Faculty of Sciences, Universidad de Chile, Santiago, Chile; 5 Center for Biomedical Research, Faculty of Biological Sciences and Faculty of Medicine, and Fondo de Áreas Prioritarias (FONDAP) “Center for Genome Regulation”, Universidad Andrés Bello, Santiago, Chile; INSERM U901, FRANCE

## Abstract

Prenatal stress causes predisposition to cognitive and emotional disturbances and is a risk factor towards the development of neuropsychiatric conditions like depression, bipolar disorders and schizophrenia. The extracellular protein Reelin, expressed by Cajal-Retzius cells during cortical development, plays critical roles on cortical lamination and synaptic maturation, and its deregulation has been associated with maladaptive conditions. In the present study, we address the effect of prenatal restraint stress (PNS) upon Reelin expression and signaling in pregnant rats during the last 10 days of pregnancy. Animals from one group, including control and PNS exposed fetuses, were sacrificed and analyzed using immunohistochemical, biochemical, cell biology and molecular biology approaches. We scored changes in the expression of Reelin, its signaling pathway and in the methylation of its promoter. A second group included control and PNS exposed animals maintained until young adulthood for behavioral studies. Using the optical dissector, we show decreased numbers of Reelin-positive neurons in cortical layer I of PNS exposed animals. In addition, neurons from PNS exposed animals display decreased Reelin expression that is paralleled by changes in components of the Reelin-signaling cascade, both *in vivo* and *in vitro*. Furthermore, PNS induced changes in the DNA methylation levels of the Reelin promoter in culture and in histological samples. PNS adult rats display excessive spontaneous locomotor activity, high anxiety levels and problems of learning and memory consolidation. No significant visuo-spatial memory impairment was detected on the Morris water maze. These results highlight the effects of prenatal stress on the Cajal-Retzius neuronal population, and the persistence of behavioral consequences using this treatment in adults, thereby supporting a relevant role of PNS in the genesis of neuropsychiatric diseases. We also propose an *in vitro* model that can yield new insights on the molecular mechanisms behind the effects of prenatal stress.

## Introduction

Stress in early life has been proposed as a main cause of neuropsychiatric diseases such as depression, schizophrenia and bipolar disorders [1–3]. Stress-related problems may have profound effects on adult brain functions which are dependent on the correct structure and operation of neuronal networks. More specifically, prenatal development is a period particularly susceptible to stress. Indeed, application of stress protocols to pregnant animals increases the release of stress-related hormones such as cortisol, which in turn induces higher than normal cortisol concentrations in embryos, which may affect the expression of other factors and in general, development and growth [4–7].

It is very likely that the effect of stress during pregnancy lasts postnatally, even through adulthood, predisposing subjects to cognitive and emotional disturbances. Animal models of prenatal stress usually include exogenous administration of glucocorticoids and restraint of pregnant dams. Regardless of the chosen method to induce prenatal stress, newborn and adult rodents display several abnormalities such as decreased number and complexity of dendritic spines in the hippocampus [8], decreased expression of mineralocorticoid receptors [9], and global changes in DNA methylation including gene promoters [10]. In adulthood, prenatal stress has been associated with anxious behavior [11, 12], decrease in synaptic plasticity [13], and impairments in learning and memory [12, 14, 15], all of which are conditions that support etiological relationships between prenatal stress and some neuropsychiatric diseases.

Some of the prenatal effects of stress are likely to involve cytoskeletal reorganization [16]. However, there are very few studies linking prenatal stress with molecules implicated in cytoskeletal modulation, including one report of a decrease in the levels of phosphorylated GSK3β, a protein kinase linked to cytoskeleton dynamics, due to maternal stress during pregnancy [17]. A critical protein in regulating brain development through cytoskeletal modifications is the extracellular matrix glycoprotein Reelin [18–20]. During development, Reelin is secreted by Cajal-Retzius (CR) neurons located principally in the cortex and the hippocampus [21], and functions via activation of a signaling cascade that involves effector proteins like mDab1 and kinases like GSK3β and Cdk5 [22, 23]. Reelin regulates the migration of neurons in laminar structures of the brain, including the hippocampus, cerebral cortex and cerebellum [20] among other regions [24], and has been proposed to play a major role in the origin and structuring of the mammalian neocortex [25, 26]. In the postnatal brain, Reelin has been identified in synaptic boutons (especially in GABAergic hippocampal neurons) and has been implicated in neuronal plasticity [27–29]. Furthermore, human post-mortem studies reveal that bipolar, schizophrenic and depressive patients display low levels of Reelin suggesting a potential role for Reelin in the etiopathology of these diseases [30–32]. Finally, down-regulation of Reelin through DNA methylation at its promoter sequence is found in stressed adult rats and in schizophrenic patients [33, 34]. Together, all this evidence places this protein and the epigenetic regulation of its expression as a likely target for the development of neuropsychiatric pathology [35, 36].

In this report, we analyzed the effects of restraint stress in pregnant rat females on the expression of Reelin and its signaling pathway in the cerebral cortex of the offspring. Moreover, anxious behaviors, memory and learning of young, prenatally-stressed rats were analyzed, thereby assessing the postnatal behavioral effects of this manipulation that are consistent with a potential correlation between reelin expression and behavioral impairments. The implications of our findings provide an important contribution to the study of the etiology of neuropsychiatric conditions.

## Materials and Methods

### Animals and restraint stress protocol

Pregnant Sprague-Dawley rats (n = 14) were housed in groups of five, under a 12-hour light/dark cycle (lights on at 7:00 A.M.) with ad-libitum access to food and water in a temperature-controlled room. Pregnant rats were randomly assigned to 2 different groups: control (n = 7) and stress (n = 7). Additionally there were no differences in reproduction activity in PNS and control dams (control (n = 10) and stress (n = 11)). Efforts were made to minimize the number of animals used and their suffering. All procedures were in accordance with the guidelines of the National Commission of Scientific Research and Technology of Chile (CONICYT) and approved by the animal care committee of the Pontificia Universidad Católica de Chile.

**Fig 1:**
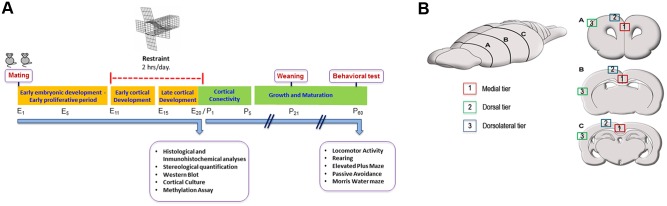
Experimental design and reference map. **A**, Experimental protocol of prenatal restraint stress. **B**, Map of neuronal quantification. The frontal (A), parietal (B), and caudal (C) cortical levels were subdivided in a medial (red square; 1), dorsal (blue square; 2) and dorsolateral (green square; 3) tiers. E = embryonic days; P = postnatal days.

Restraint stress was performed between the embryonic days 11 and 20, as described by Vyas et al. [37]. The prenatal restraint stress (PNS) group of pregnant rats suffered restraint once a day for 2 hours (from 12 P.M. to 2 P.M.). The dimensions of the rodent restraint cages were: length 18 cm, width 6 cm and height 6 cm. The cage enables complete restraint of animal subjects without obstructing normal breathing; furthermore, they may urinate and defecate without being in contact with their waste. Unstressed controls were first handled and then left undisturbed in their home cage. The weight gain was measured from day 10 until day 20. Fig. 1A outlines all procedures presented as a time-line.

### Tissue preparation

At gestation day 20, we randomly chose 4 pregnant dams from each group for histological and biochemical analysis. Remaining rats were maintained until they gave birth, and their offspring were kept for future behavioral tests. The chosen pregnant rats (Control = 4, PNS = 4) were euthanized by an overdose of sodium pentobarbital, and perfused transcardially with 0.9% saline. At the time of perfusion, adrenal glands and fetuses were removed. Adrenal glands were weighed. E20 fetal brains were hemisected and random brain hemispheres were fixed overnight in 4% paraformaldehyde and cut in coronal sections using a vibratome (Leyca VT 1000S) (Control = 30, PNS = 30). Sections of 70 μm were obtained and maintained in phosphate-buffered saline (PBS), pH 7.4 at 4°C until processing for immunohistochemistry. The remaining unfixed brain hemispheres were maintained at -80°C and randomly used for protein extraction or cortical neuron culture.

### Immunohistochemistry

For Reelin and NeuN immunohistochemistry, frontal, parietal and retrosplenial brain sections of each fetus were selected (Fig. 1B). Endogenous peroxidase of free-floating sections was inactivated twice for 15 min at room temperature (RT) with H_2_O_2_ at 6% in PBS. After 3 washes with PBS, sections were immersed in blocking solution (IHC Select Detection Systems, Millipore) for 2 hours at RT, and then incubated with the monoclonal anti-Reelin (1:1000, Millipore) and anti-NeuN (1:100, Millipore) as primary antibody, over 2 nights at 4°C. After the incubation and the corresponding washes with PBS, brain sections were incubated for 1 hour at RT with the biotynilated anti-mouse secondary antibody (IHC Select Detection Systems, Millipore), then and after subsequent washes with PBS, sections were incubated with Streptavidin-HPR (IHC Select Detection Systems, Millipore) for 1 hour at RT. After washing, the staining was revealed using diaminobenzidine (1:400, IHC Select Detection Systems, Millipore). Finally, sections were cleaned in distilled water and mounted.

### Stereological quantification

Reelin and NeuN immunoreactive neurons were quantified in 9 different cortical regions distributed along the anteroposterior and mediolateral axes of the rat brain (Fig. 1B)[38]. Observations were made at three different rostrocaudal levels (frontal, parietal and retrosplenial) in coronal sections. In each of these levels, we took samples from medial, dorsal and dorsolateral tiers (Fig. 1B). More specifically, at the frontal level we took samples from the medial tier, including the prospective of the adult anterior precentral and cingulate areas of the prefrontal cortex, from the dorsal tier including the early location of the motor cortex and from the dorsolateral tier including early somatosensory cortex (Fig. 1B, section A). At the parietal level, the cortex was subdivided into a medial tier including the future cingulate and motor cortex, and dorsal and dorsolateral tiers, both including early somatosensory cortex (Fig. 1B, section B). More posteriorly, the caudal level was subdivided into a medial tier including the posterior cingulate cortex, a dorsal tier including the posterior parietal cortex and a dorsolateral tier including the maturing auditory cortex (Fig. 1B, section C). In each region, stereological quantification was performed using the optical dissector method as described by Cruz-Orive and Weibel [39]. The counting frame of each dissector square had dimensions of 0.08 × 0.08 mm and the height was 0.01 mm. Double-blind counting was performed in 5 randomly selected dissector fields out of 10 in the first layer of all considered areas, which correspond to the length of one dissector square. The final neuron number was determined with an average of both blind-counts and considering a dissector volume of 6,4 × 10^-5^ mm^3^. Observations were made with bright field microscopy, and the images were captured using an Olympus microscope (Model BH-2, Olympus Co., Tokyo, Japan) equipped with a Moticam 2500 photographic camera (Speed Fair Co., Ltd).

### Cortical culture

Cerebral cortex neurons were dissociated mechanically in cold PBS 1X and incubated with HBSS—EDTA Trypsin 1%(10:1) for 30 minutes. Then, cells were dissociated in HBSS by gentle sweeping, counted and seeded into poly-D-lysine-coated dishes in DMEM 10% HS. Finally and after 2 hours of incubation, plated medium was changed by neurobasal medium supplemented with B27, glutamine 2mM, pyruvate 1mM, 100 μg/mL of penicillin and 100 μg/mL of streptomycin for 5 days *in vitro* at 37°C, 95% humidity and 5% of CO2.

### Western blot analysis

After RIPA culture and tissue protein extraction, 30 μg of protein were loaded in a 6%, 10% or 12% polyacrylamide gel (according to the molecular weight of the protein analyzed). Samples were run in the concentrator gel for 30 min at 80 V and separated at 120V at different times, depending on the molecular weight of the protein of interest. Proteins were transferred to a nitrocellulose membrane using a wet protocol for 1.5 hour at 330 mA for proteins displaying high molecular weights, and for 1 hour at 300 mA for proteins displaying small molecular weights. Membranes were then blocked using non-fat dry 5% milk in TBS 1X, TWEEN 0,05% and incubated with primary antibody in non fat dry 1% milk in TBS 1X, TWEEN 0,05% over night at 4°C (anti-Reelin 1:1000, Millipore; anti-mDab1 1:1000, Santa cruz; anti-p35 1:1000, Santa cruz; anti-CDK5, 1:1000, Santa cruz; anti-PHF-1. 1:3000, donated by Peter Davis, Albert Einstein College of Medicine, NY, USA; anti-Tubulin 1:10000, Sigma). Membranes were then washed six times with TBS 1X, TWEEN 0,05% for 6 min and incubated with secondary antibody for 2 hours in non fat dry 1% milk TBS 1X, TWEEN 0,05% at room temperature (anti-MOUSE 1:5000 Jackson; anti-RABBIT 1:5000 Jackson). Finally membranes were washed five times for 6 min in TBS 1X, TWEEN 0,05%, and the presence of proteins was developed using an ECL kit (Pierce Thermo Scientific) and radiographic films (Fujifilm).

### DNA Methylation assay

Whole cerebral cortex and cultured cortical neurons derived from newborn rats were collected for genomic DNA extraction. Firstly, cells were washed three times with 1X PBS, placed on ice, collected and centrifuged at 2,000 g. Cell pellets were resuspended in digestion buffer (100 mM NaCl, pH 8.0 Tris-Cl 10 mM, pH 8.0, 25 mM EDTA, 0.5% SDS, proteinase K 0.1 mg / ml) and incubated overnight at 40°C. Genomic DNA was isolated using phenol/chloroform extraction and finally resuspended in TE 1X buffer pH 8.0 and quantified by spectrophotometric methods. Two μg of purified genomic DNA were digested with HpaII or MspI restriction enzymes (sensitive and insensitive to DNA methylation, respectively). Whole cerebral cortex DNA samples were obtained by pre-digesting the samples with EcoRI (the DNA region analysed in this study does not contain such restriction site). This procedure further increases solubility of the DNA samples and therefore facilitates subsequent quantification and cleavage with HpaII/MspI. Conventional PCR was performed using 200 ng of digested genomic DNA and specific primers against the Reelin gene promoter as well as against other genomic regions used as controls (Table 1). Finally, PCR amplification products were analyzed by electrophoresis in 1% agarose gels [40].

**Table 1:** Primers used in each condition.

Reelin	Forward	5′-GGAAACGCATTAAAACCTG-3′
	Reverse	5′-GGAAGGCATGCAGAGGAGT-3
Runx2	Forward	5′-GTGGTAGGCAGTCCCACTTT-3′
	Reverse	5′-TGTTTGTGAGGCGAATGAAG-3′
Ric8B	Forward	5′-TGGTTTCCGGCCTTTAGGGAAC-3′
	Reverse	5′-GGAGCCACCAGAGACTGAGTCA-3′

### Cdk5 kinase activity assay

Cdk5 activity was measured as described previously [41]. Briefly, 300 μg of protein were extracted from brain cortex of control and stressed rats. Proteins were dissolved in T-PER buffer and immunoprecipitated using 4 μg of anti-Cdk5 antibody C8 (Santa Cruz, CA). Immunoprecipitated proteins (IP) were washed 3 times in cold PBS, and 2 times in kinase buffer [20 mM Tris HCl (pH 7.4), 10 mM MgCl2 and 1 mM EDTA]. IP were then mixed with the kinase assay mixture [100 mM Tris • HCl (pH 7.4), 50 mM MgCl2, 5 mM EDTA, and 5 mM DTT] plus 5 μCi (γP32)-ATP, with 5 μg of Histone H1 used as a substrate. Kinase assays were carried out at 30°C for 30 min and the kinase activity reaction was stopped by adding 5xSDS sample buffer and boiling it for 10 min at 95°C. The kinase reaction was electrophoresed on a 12% polyacrylamide gel and then gels were exposed to X-ray films for 1–3 h at −80°C. The incorporation of P32 to Histone H1 was quantified to measure band intensity using ImageJ 1.46r software (NIH, MD).

### Behavioral activity

All behavioral procedures were performed with rats aged two months (P60), considered as young adults. Sprague—Dawley rats, weighing 200–230 g, were housed eight per cage in a temperature-controlled animal facility, under a 12:12 hour light—dark cycle (lights on from 0800 to 2000 hr) with food and water *ad libitum*. Behavioral observations took place in a soundproof room at the same time of the day (10:00 to 12:00 hr) to reduce the confounding influence of diurnal variation on spontaneous behavior. Female rats were tested in diestrus, to avoid fluctuations in the data due to effects of the cycle itself [42]. For data analysis of locomotor activity, rearing, elevated plus maze and Morris water maze, 10 animals were used for each treatment (5 males and 5 females). In the passive avoidance paradigm we used 9 animals for each treatment, with 5 females and 4 males in each group. Each animal was evaluated only once on each behavioral test.

### Spontaneous Motor activity

Each rat was placed in a plexiglass cage (30 × 30 × 30 cm), and spontaneous locomotor activity and rearing were monitored during a period of 30 min and evaluated as described previously [43]. The floor of the cage was an activity platform (Lafayette Instrument Co., Lafayette, IN, USA) connected to an electromechanical counter. In order to avoid the influence of disturbing noises, the platform was placed in a soundproof chamber. Each animal was observed continuously via a Sony video camera connected to a VHS tape recorder (Sony Corporation, Mexico DF, Mexico). Scores were generated from live observations, while video sequences were used for later re-analysis when necessary. Results were expressed as the score measured on 30 min of observation for each group.

### Elevated plus-maze

Following the analysis of spontaneous locomotor activity and rearing behavior, anxiety levels were measured using the elevated plus-maze test. Each rat was individually placed in an elevated plus-maze consisting of two open arms (50 × 10 cm each), two closed arms (50 × 10 × 20 cm each) and a central platform (10 × 10 cm), arranged in such a way that the two arms of each type were opposite to each other. The maze was elevated 100 cm above the floor. At the beginning of each trial, animals were placed at the center of the maze, facing a closed arm. During a 5 min test period, we recorded: (i) the number of open arm entries; (ii) the number of closed arm entries; (iii) the time spent in open arms; and (iv) the time spent in closed arms. Entry into an arm was deflned as the animal placing all four limbs onto the arm. The maze was thoroughly wiped clean with 5% ethanol solution after each trial. All trials were conducted between 10:00 and 14:00 hours. Results were expressed as percentages of time spent in open arms and the total number of entries (sum of closed and open arm entries) on 5 min of observation for each group.

### Learning and memory tasks

**Passive Avoidance Conditioning**. The test was carried out in a two-way shuttle box (Lafayette Instrument Co, IN, USA) composed of two stainless steel modular testing units with a manual guillotine door placed between them. Each modular chamber was equipped with an 18 bar insulated shock grid connected to a shocker (Master Shock Supply, Lafayette Instrument). One of these chambers remained illuminated and the other was darkened. On day 1 of testing, animals were habituated to the apparatus. Each rat received two trials with an interval of 20–30 min. On each trial, the rat was placed into the illuminated chamber facing away from the guillotine door. When the animal entered the darkened chamber, the guillotine door was lowered noiselessly and the animal was removed from the apparatus 10 sec later. The latency to enter was recorded. On day 2, each animal was placed into the illuminated chamber and permitted to enter the darkened chamber. Upon entry to the darkened chamber, the guillotine door was lowered and a 0.35 mA foot shock was applied for 2 sec through the grid floor. 10 seconds after this training, the rat was removed from the apparatus. The retention test was given on day 3 and consisted of a single trial without foot shock, in which each animal was placed into the illuminated chamber and the latency to enter the darkened chamber was recorded to an arbitrary maximum of 300 sec. On days 4 and 5, the extinction of the response was assessed, in a way similar as that on day 3. This test is based on the conflict between unconditioned avoidance of a brightly lit chamber and conditioned avoidance of an electric shock. Results are presented as the median latencies, which served as an index of retention and extinction on each day for each group.

**Morris water maze test**. The water maze test used in our experiments was described by Morris in 1984 (Morris, 1984). This method is used to assess spatial learning and memory in rodents and it was performed at P60. The apparatus consists of a circular swimming pool (200 cm diameter, 60 cm depth) that was filled with water (22° C temperature) to 20 cm below the rim. The pool was divided into four quadrants of equal area (designated north, south, east, and west. A circular Plexiglas platform (10 cm diameter) was hidden just 2 cm below the water surface in the middle of the north quadrant. This platform had the same color as the swimming pool in order to render it invisible to the rat. The procedure room, where the swimming pool was located, had distinctive visual cues on the walls for spatial orientation. Rats were tested individually and placed into various quadrants of the pool. The time elapsed and the distance traversed to reach the hidden platform was recorded. Rats were moved to the procedure room 30 min before testing, and each animal was put through five trials per day, with 20-min intertrial intervals. The two investigators that worked with the water maze procedure were always the same and located always at the same position in the room. On each trial the rat was placed into the water, facing the side walls of the pool at one of the cardinal compass points (north, south, east, or west), and was allowed to locate the platform with a maximal time of 120 sec. The time spent by the rat to find the platform was recorded and assigned as latency. If the rat failed to find the platform within the maximal time, it was guided manually to the escape platform and allowed to remain there undisturbed for 30 sec. In this case it was assigned a latency of 120 sec. The sequence of starting quadrants was counterbalanced every trial. After staying on the platform for 30 sec, the rat was gently picked up, returned to its home cage, and allowed to warm up and dry off under a heat lamp. The entire procedure took 5 days and results were expressed as the time in seconds of latencies in swimming performance on each day for each group.

### Statistics

For all treatments, we measured the effect of PNS treatment compared with control animals. Values are expressed as the mean ± SEM. For the analyses of the Reelin immunohistochemistry quantification and Western Blot, adrenal gland, and body weight gains we used the t-test (unpaired and Mann-Whitney test) considering p<0.05 as statistically significant. Western Blot quantification was done with ImageJ program. In the behavioral anxiety data (locomotor activity, rearing and elevated plus maze) we used the t-test (unpaired) considering p<0.05 as statistically significant. For the passive avoidance and water maze analyses, two-way analysis of variance (ANOVA) was used. Post-hoc comparisons between groups were made using Tukey’s o Bonferroni test. To assess differences in behavior between males and females we used the Mann-Whitney non-parametric test. All gender comparisons yielded nonsignificant differences (P> 0.07). For all tests, the value p<0.05 was considered significant. The statistics and the graphs were made with GraphPad Prism 5.

## Results

### Prenatal restraint stress decreases Reelin-expressing neuron density in E20 embryo brains

**Fig 2:**
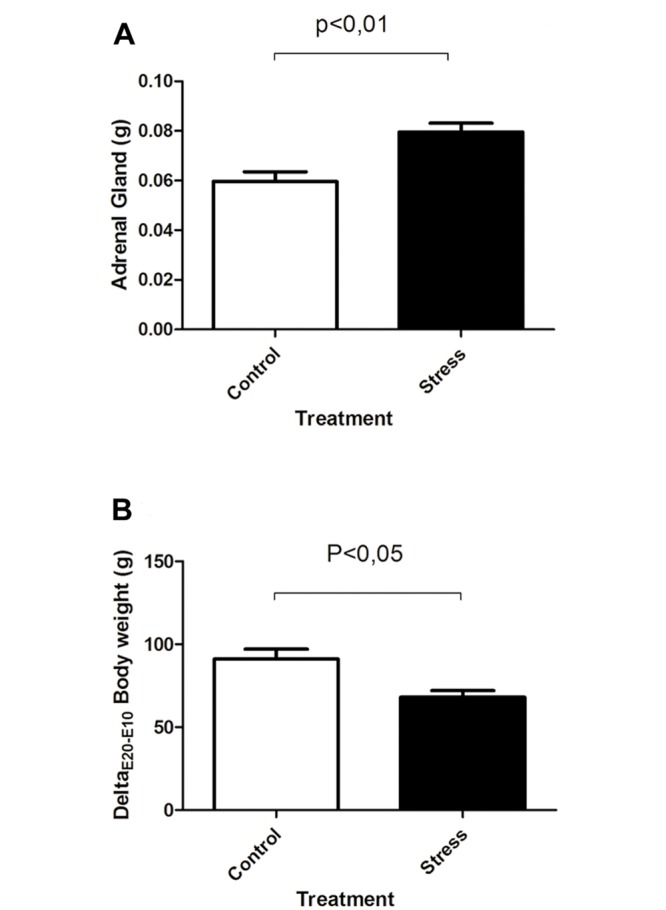
Validation of stress in the pregnant rat. **A**, The average adrenal gland weights were higher in stressed dams with respect to controls. **B**, analogously, in stressed rats the average body weight gain was 22.8% less than in controls. Values are mean ± SEM, n = 7 rats per group.

Pregnant rats were restrained, from E11 until E20, for 2 hours per day. Generation of stress in pregnant rats was confirmed by an increase in adrenal gland and body weights, between days E11 and E20. We observed that the average adrenal gland weight was 25% higher in stressed rats compared to controls (p<0.05; Control: 0.0596 ± 0.003g, n = 7; PNS: ± 0.07958 ± 0.003, n = 7) (Fig. 2A). Similarly, stressed rats showed that the average body weight gain was 22,8% lower than in controls (p<0.05; Control: 89.19 ± 4.42g, n = 7; PNS: ± 68.75 ± 3.5, n = 7) (Fig. 2B). These two parameters confirm that our restraint protocol generates stress in pregnant rats.

**Fig 3:**
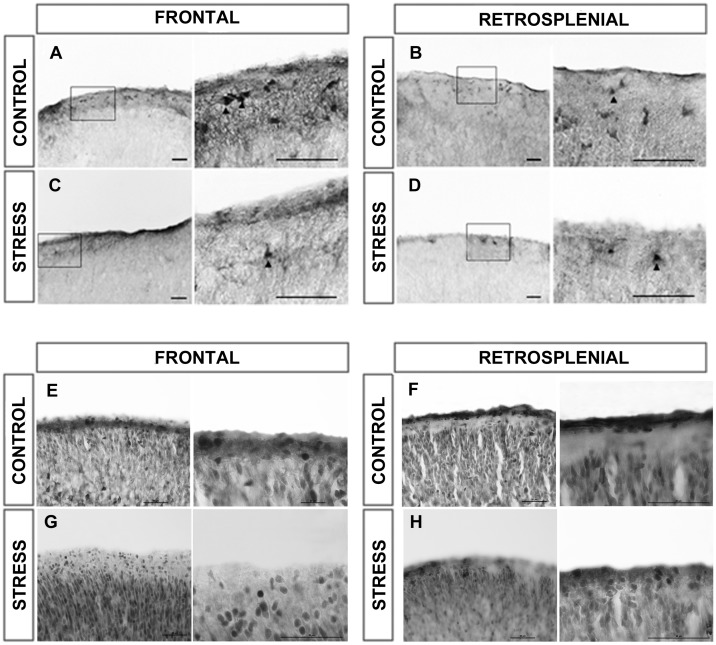
Cerebral cortex reelin and NeuN immunohistochemistry. Microphotographies of the dorsal tier of frontal (**A, C, E, G**), and retrosplenial (**B, D, F, H**) cortical levels. Control brains (**A, B**) show numerous clusters of Cajal-Retzius neurons while the stress groups (**C, D**) show only few isolated Cajal-Retzius neurons. Total neuronal density is show in Control brains (**E, F**) and stress group (**G, H**). No differences between groups are observed. Scale bars = 50 μm.

We counted Reelin positive neurons present in the medial, dorsal and dorsolateral tiers at frontal, parietal and retrosplenial levels. We focused our attention in the subset of neurons intensely stained in the cortical layer I (marginal zone), as shown in Fig. 3. Control sections display Reelin positive neurons in cortical layer I which as expected are arranged parallel to the cortical surface (Fig. 3A, B). In PNS rats there is a decreased number of Reelin positive neurons, which are sparsely distributed within the marginal zone, although they remain aligned and parallel to the cortical surface (Fig. 3C, D).

In order to evaluate whether the decrease in Reelin-positive neurons was due to cell death, we performed a NeuN quantitative analysis of the cortical layer I (Fig. 3 E, F, G, and H). Although we did not specifically control for CR cells using markers different than Reelin, these results indicate that the total number of neurons was conserved in the experimental group, suggesting that the decrease in Reelin protein was due to a deficit in gene expression.

**Fig 4:**
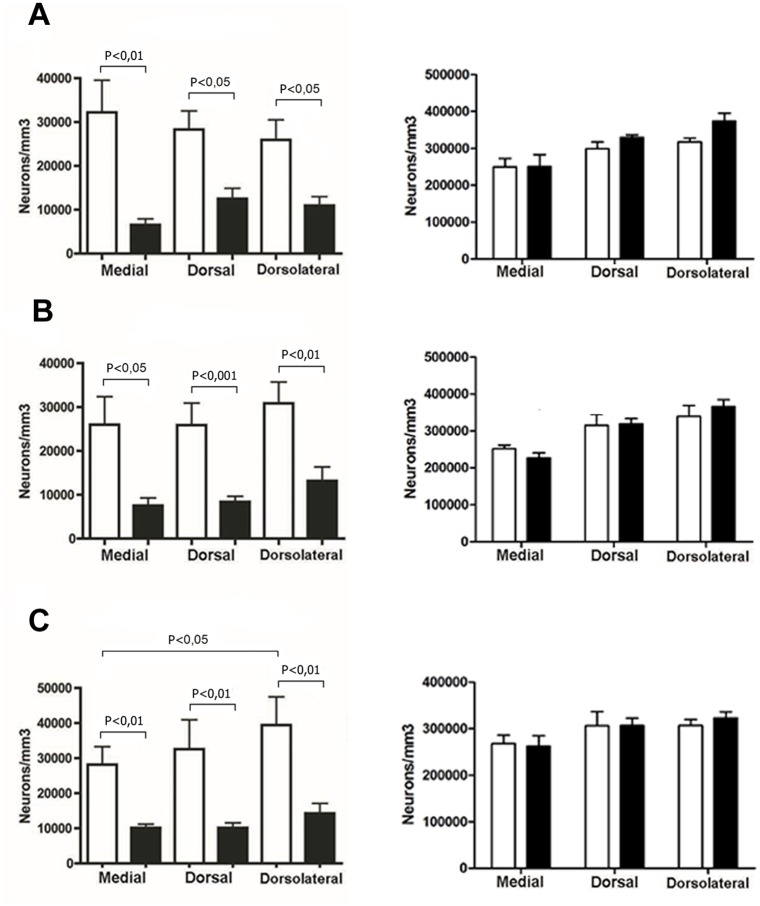
Prenatal stress induces global decrease of reelin expressing neurons density in the prenatal (E20) rat brain (Left side) and no changes of NeuN neurons density (Right side). Immunohistochemistry results: All graphs show the immunoreactive neurons expressed in neurons/mm3. Opens bars represent control group and filled bars represent stress group. **A**, **B** and **C**, show the results of frontal, parietal and retrosplenial cortex respectively. Values are mean ± SEM, n = 7–10 rats per group (reelin analysis) and n = 3 rats per group (NeuN analysis).

Quantitative analysis of E20 embryo brains confirms that there is a significant decrease in Reelin staining at CR neurons in rats who suffered PNS, respect to control, in all analyzed areas (Fig. 4, Left). The prefrontal cortex was the most affected area, exhibiting a decrease of 79% in Reelin immunoreactivity (p<0.01, n: Control = 8, PNS = 6) (Fig. 4A, Left). The dorsolateral parietal cortex was less affected, with a decrease percentage of 58% (p<0.01, n: Control = 8; PNS = 7) (Fig. 4b, Left). Similarly, we found significant differences at the retrosplenial level in the control group, where Reelin positive neurons are 28% more abundant in the dorsolateral (auditory) cortex respect to the medial (retrosplenial) cortex (p<0.05, n = 6) (Fig. 4C, Left). No differences were found between stress and control conditions at any of the analyzed regions (Fig. 4A–C, Right).

### Changes in the Reelin signaling pathway using *in vivo* and *in vitro* PNS models

**Fig 5:**
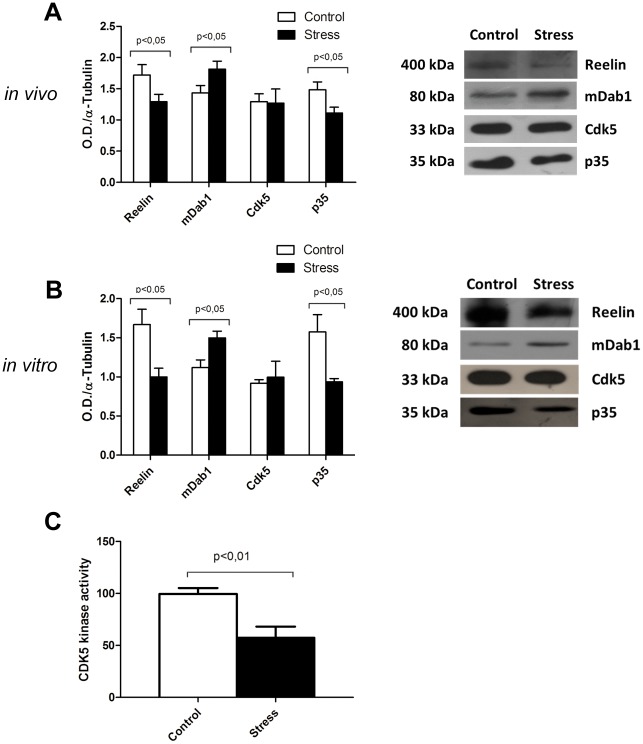
*In vivo* (A) and *in vitro* (B) expression of Reelin and downstream elements in control and stress conditions analyzed by Western Blot. The left side of the panel shows the result of density pixel quantification, the right side shows a representative example of each protein expression. Expression levels observed *in vivo* are maintained after 5 DIV. Values are mean ± SEM. n = 8–12 per group at *in vivo* experiments, n = 3 per group at *in vitro* experiment. (**c**) Cdk5 kinase activity measured from protein extract from brain cortex of control (n = 5) and stressed (n = 4) rats. All data are presented as the mean and SEM. * p < 0.05.

Having shown that PNS induced significant changes in the amount of Reelin expression in cortical neurons, we determined whether these changes in Reelin levels are paralleled by alterations in the Reelin-dependent signaling pathway. For this purpose, we analyzed by Western Blot the expression of Reelin and Reelin-dependent signaling components in cortex protein extracts. We found that the overall expression of Reelin decreases in PNS pups as compared with control animals (p<0.05; Control: 1.719 ± 0.1671, n = 12; PNS: 1.292 ± 0.1203, n = 12) (Fig. 5A), confirming our previous immunohistological results. Furthermore, PNS pups displayed increased levels of the adaptor protein mDab1 with respect to controls (p<0, 05; Control: 1.434 ± 0.1203, n = 12; PNS: 1.815 ± 0.1286, n = 15) (Fig. 5). We also determined the overall levels and activity of the protein kinase CDK5 and its activator (p35), as read-outs for the Reelin signaling pathway. The protein kinase levels (Control: 1.294 ± 0.1266, n = 8; PNS: 1.269 ± 0.2275, n = 8) remained unaffected, while p35 concentrations decrease in PNS compared to controls (p<0.05; Control: 1.484 ± 0.1254 n = 8; PNS: 1.114 ± 0.09210 n = 8) (Fig. 5A). As the activity of Cdk5 has been shown to be limited by the availability of p35 in neurons, we analyzed Cdk5 activity in control and PNS brain extracts. Fig. 5C shows that there is a decreased phosphorylation of histone H1 (p<0.01; Control: 100 ± 5.45, n = 5; PNS: 57.66 ± 10.84, n = 4), a canonical readout for Cdk5 activity, suggesting that decreased Reelin signaling negatively impacts this kinase activity (Fig. 5C). As a complementary approach we cultured cortical neurons from PNS and control embryos over 5 days, and repeated the analysis of molecular markers for the Reelin-signaling pathway. As shown in Fig. 5B, our previous findings were confirmed in cortical neuron cultures; Reelin (p<0.05; Control: 1.669 ± 0.1942, n = 3; PNS: 1.001 ± 0.1098, n = 3) and p35 levels (p<0.05; Control: 1.577 ± 0.2200, n = 3; PNS: 0.9370 ± 0.04292, n = 3) are lower in PNS subjects (p<0.05, n = 3), while mDab1 is higher in PNS than controls animals (p<0.05; Control: 1.119 ± 0.09797, n = 3; PNS: 1.499 ± 0.08474, n = 3). In contrast, CDK5 kinase protein levels remained unaffected (Control: 0.9197 ± 0.04484, n = 3; PNS: 0.9951 ± 0.2060, n = 3) (Fig. 5B). Altogether these results confirm that PNS-induced down-regulation of Reelin expression is paralleled by decreased Reelin-dependent signaling both in brain and cortical neuron primary cultures.

### Methylation of the Reelin gene promoter is increased in the cortex of PNS new born rats

**Fig 6:**
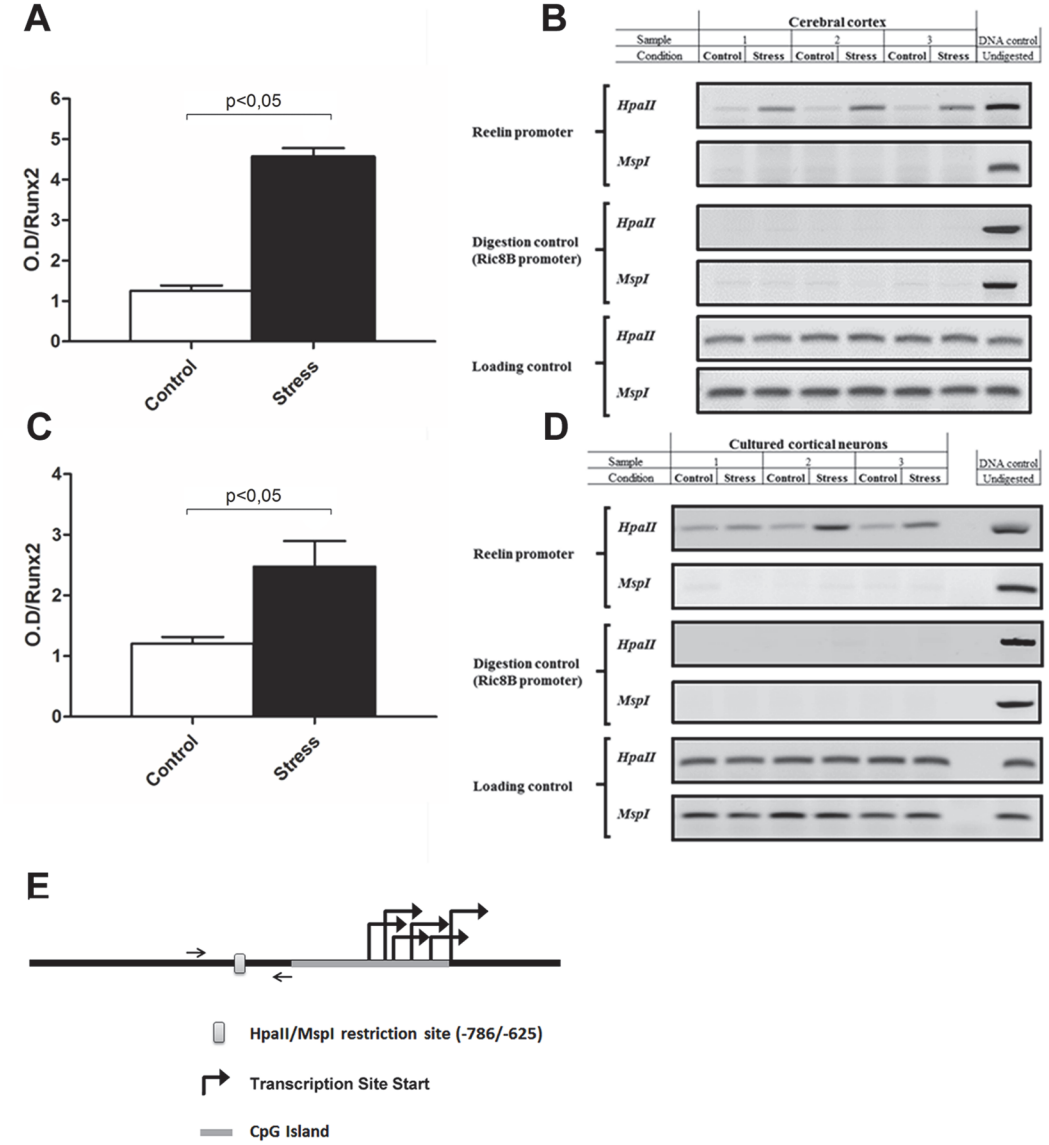
*In vitro* (A) and *in vivo* (C) analyses of DNA methylation at the reelin gene promoter region in samples from control and stress groups. Genomic DNA obtained from both groups was cleaved with DNA methylation sensitive restriction enzyme HpaII and then amplified by conventional PCR using specific primers against a reelin promoter region containing an HpaII site (-786/-625). Undigested genomic DNA is included as amplification control in all PCR reactions. Efficient digestion of DNA samples using the HpaII enzyme was controlled by analyzing a region devoid of DNA methylation at the Ric-8B gene promoter containing several HpaII cleavage sites (digestion control). A region of the Runx2 gene promoter lacking HpaII sites was selected to show equal sample loading in each lane of the gels. Digestion with the isoschizomer restriction enzyme MspI (DNA methylation insensitive) was also performed to control for efficient cleavage at the reelin promoter sequence analyzed (Data not shown). DNA methylation differences between control and stress rats groups measured *in vivo* (**B**) and *in vitro* (**D**) were quantified by determining changes in pixel density at the bands amplified by PCR and visualized through conventional DNA electrophoresis (see methods). (**E**), diagram depicting the selected upstream promoter region with its putative methylation sites. The reelin promoter has several transcription site starts over the CpG island. Upstream the promoter is the HpaII/MspI restriction site that was selected for our analyses. Values are expressed as relative ratios between PCR amplifications of the reelin promoter sequence after digestion with HpaII over the amplifications of the Runx2 control promoter sequence (loading control). Values represent a mean ± SEM. N = 3 per group. *p<0.05.

Because stress can change gene expression by modifying epigenetic control mechanisms we next addressed whether our PNS protocol down-regulates Reelin expression through changes in its promoter’s CpG methylation status. We performed a DNA methylation detection analysis by measuring cleavage by specific restriction enzymes followed by PCR amplification using primers against the Reelin promoter region. The Reelin promoter contains mainly two regulatory regions that are proximal and distal to the transcription start site. We focused our analyses on the distal promoter region that contains a CCGG site that can be target for cleavage by the HpaII/MspI restriction enzyme pair (Fig. 6E). In genomic DNA extracted from fresh cerebral cortex, HpaII cleavage at this Reelin regulatory region showed significant differences between PNS and control groups, indicating that PNS treatment results in increased CpG methylation (Fig. 6A, B; p<0.05; Control = 1.252 ± 0.1372 n = 3; PNS = 4.569 ± 0.2143 n = 3). Similar results were obtained when DNA samples isolated from primary cultures enriched in cortical neurons grown *ex vivo* were analyzed (Fig. 6C, D; p<0.05; Control: 1.207 ± 0.1095 N = 3; PNS: 2.477 ± 0.4217 N = 3). Together, these results indicate that down-regulation of Reelin expression in PNS neurons is strongly associated with increased CpG methylation at the promoter region of this gene.

### PNS rats display anxiety like behavior

**Fig 7:**
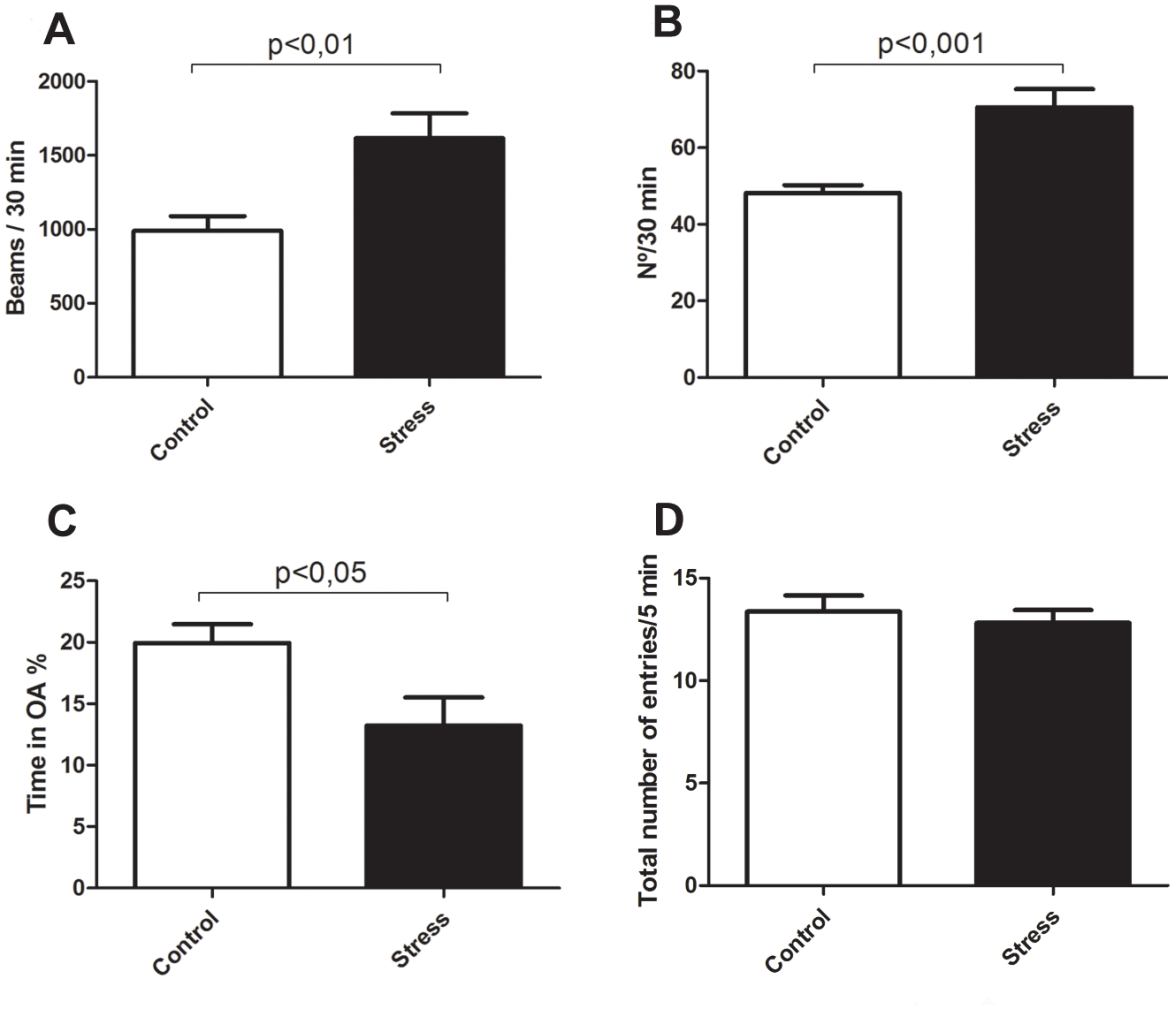
Prenatal stress increases the spontaneous rearing (A) and spontaneous locomotor activity (B) responses in young adult rats (P60). Values are mean ± SEM, n = 11 rats per group. **C** and **D**, Prenatal stress decreases the total time in the open arms and does not affect the total number of entries of open and closed arms in the elevated plus-maze. Values are mean ± SEM, n = 11 rats per group.

We next addressed whether our PNS protocol induces behavioral effects in young adult rats. First, Fig. 7A shows the effects of the prenatal restraint stress on spontaneous motor activity. The analysis confirms that PNS increases locomotor activity of rats (p<0.01; Control: 988.54 ± 99.94, n = 11; PNS: 1615.63 ± 169.06, n = 11). Second, Fig. 7B shows the effect of PNS on rearing activity. PNS animals exhibit increases in the rearing activity respect to controls (p<0.001; Control: 48.18 ± 2.07, n = 11; prenatal restraint stress: 70.54 ± 4.78, n = 11). Third, as shown in Fig. 7C, PNS rats spend less total time than controls in the open arms of the elevated plus-maze (p<0.05; Control: 19.95 ± 1.50, n = 11; PNS: 13.21 ± 2.29, n = 11). Fourth, Fig. 7D shows the total number of open and closed arm entries and shows no significant difference between groups (Control: 13.4 ± 0.8, n = 11; PNS: 12.1 ± 1.1, n = 11). Hence, our evaluation extending 5 minutes indicates that rats of the PNS group spent less time in the open arms position than rats of the control group even though both groups had a similar number of total entries in the maze. All gender comparisons yielded nonsignificant differences (P> 0.07) in all tasks performed.

### Emotional memory is affected in PNS rats independently of visuo-spatial memory

**Fig 8:**
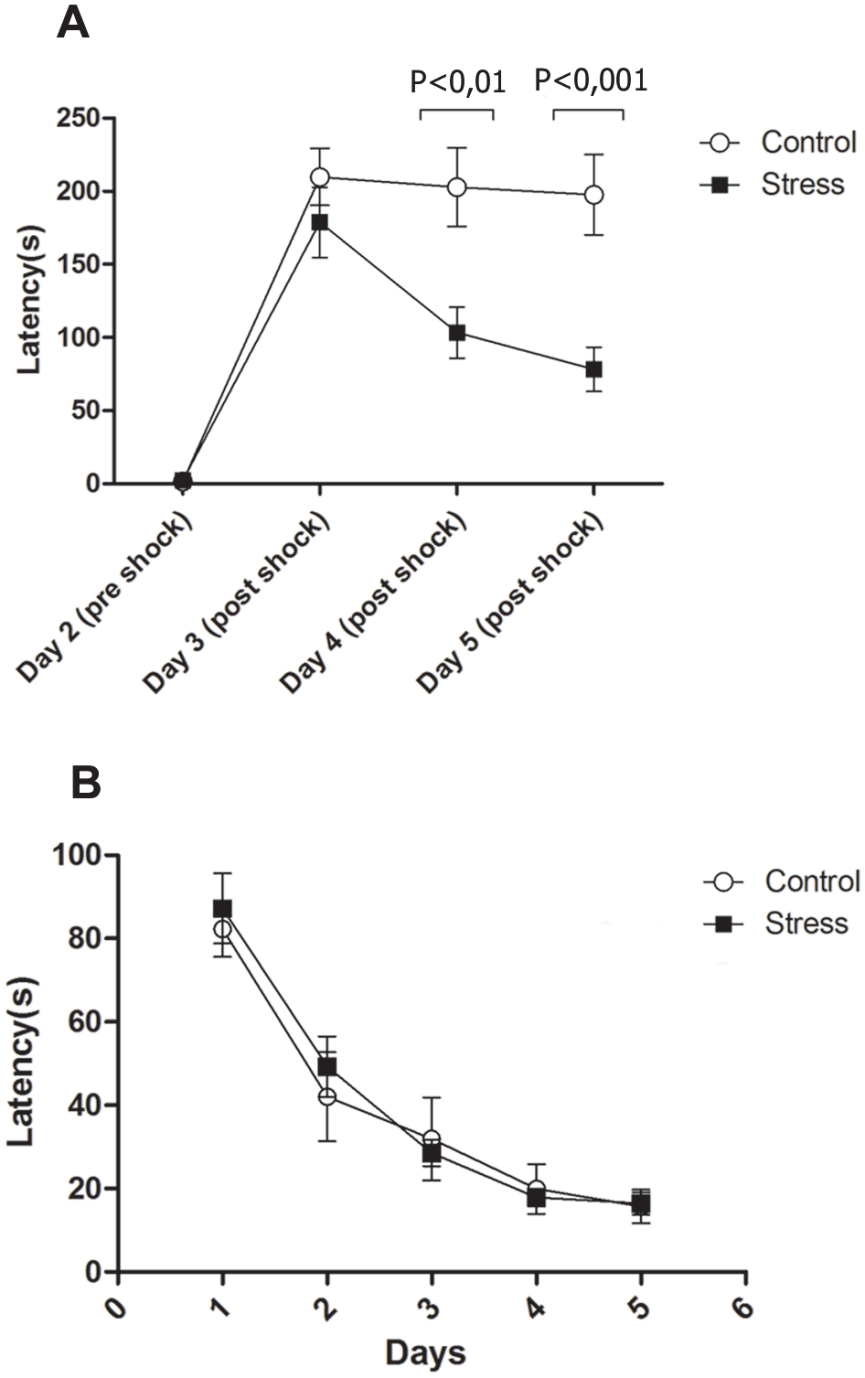
**A**, Passive avoidance learning retention test in rats. There were significant differences in latency time between control and prenatally stressed rats, on days four and five post shock. **B**, Morris maze test in rats. There were no significant differences in latency time between control and prenatally stressed rats. Each point represents mean ± SEM, n = 9 rats per group.

We also performed two memory tests; one that involves memory and learning associated with an aversive situation and the other that involves visuo-spatial memory, namely ‘passive avoidance’ and Morris tests, respectively. Fig. 8A shows the results of the passive avoidance experiment. Two-way ANOVA analysis confirms that the offspring of PNS mothers responded differently with respect to controls (F1, 16 = 9.537, p< 0, 01); in addition, the behavior on observation days was different (F3.16 = 61.83, p<0,001). Significant changes were also observed in the retention of emotional memory in PNS rats. The behavior over time of observation depends on treatment (treatment × time) (F (3.16) = 7.126, p< 0,01). Post-hoc comparisons between groups showed significant differences on Day 4 (p<0,01) and 5 post-shock. (P<0,001). All gender comparisons yielded nonsignificant differences (P> 0.36) in this task In contrast, during the Morris maze test, PNS and control groups exhibited an identical latency score on all days analyzed (Fig. 8B). Thus, both groups improved their performance over the course of the learning phase as revealed by a decrease in the latency (ANOVA; p > 0.05). These analyses indicate that the control and PNS groups did not evolve differently over the course of learning.

## Discussion

In the present study, we evaluated the effect of PNS on perinatal expression (E20) of the Reelin protein in the rat cerebral cortex. Additionally, we demonstrate the long-term behavioral effects of PNS in both genders. To our knowledge, this is the first study reporting the effects of PNS on CR neurons and Reelin expression. Firstly, we observed a decrease in Reelin immunoreactivity in layer 1 in all areas of the cerebral cortex analyzed while total neuronal numbers, as assessed by NeuN-positive neuronal counts, were not affected by the treatment. Secondly, we determined PNS effects in Reelin expression and in components of its signaling pathway, both *in vivo* and *in vitro* by Western blot, including the analysis of methylation of the Reelin promoter in both conditions. Lastly, we observed general anxiogenic effects, in addition to changes in the performance of some cognitive tasks involved in memory and learning.

PNS causes a density decrease of CR reelin-positive cells in the first layer of cortex. This effect can be the result of increased neuronal death, down regulation of the reelin gene transcription, or both. Recently, Baquedano et al. [44] generated a model that relates prenatal restraint stress to apoptosis. According to these authors, prenatal stress decreases the activation of intrinsic apoptosis through the activation of IGF-1, which stimulates Bcl-2, causing a decrease of p53 and Caspase 8 levels. On the other hand, it has been previously shown that Reelin expression can be negatively regulated by methylation of its promoter in rat offspring under a maternal absence stress protocol [34] as well as in human schizophrenic patients [45].

Additionally, it is well established that stress increases the activity of the hypothalamo-pituitary axis, increasing glucocorticoid discharge, which can cross the placenta and the blood-brain barrier [5] to generate changes in embryonic gene expression [10]. Recently, in two consecutive reports, Lussier et al. demonstrated that after 21 days of corticosterone injections, the number of Reelin positive neurons decreases in rat hippocampus [46]. Moreover, these authors exposed heterozygous reeler mice to increasing concentrations of corticosterone for 21 days, which caused an increase in depression-like behaviors, decreases in Reelin expression and in hippocampal neurogenesis, all with cell maturation impairments [47]. Other analyses have revealed that overexpression of Reelin in mutant mice eliminates the depressive-like behavior produced by corticosterone treatment [35]. On the other hand, it has been suggested that an increase in brain-derived neurotrophic factor (BDNF), modulated by stress [48–50], can affect the correct cortical lamination, by a Reelin-independent mechanism [51]. However, Ringstedt et al. [52] demonstrated that BDNF protein increases between days P0–P18, and that this increase is accompanied by a decrease of Reelin-expressing cells, thereby suggesting that BDNF plays a role in the degeneration of CR cells. Finally, Alcántara et al. concluded that BDNF modulates the spatial organization of CR and GABAergic cells in the marginal zone [53]. Putting together these previously reported findings and in light of the results communicated in this study, we can hypothesize that the differences in the Reelin expression observed in our PNS model are mainly due to down regulation of reelin gene transcription rather than to an increase in neuronal death. However, and despite our results using NeuN quantification as an indicator of the total neuronal density of layer I (Fig. 4, Right), an additional apoptosis assay may be required to confirm this possibility.

Our *in vitro* and *in vivo* DNA methylation analyses support the idea that stress can epigenetically down-regulate the expression of Reelin in cortical neurons. It is important to remark that the procedures used to demonstrate the effects of PNS on Reelin expression are complementary to those used to evaluate methylation, as they involve different resolution levels. While immunohistochemistry and western blot are techniques that mainly consider Reelin producing neuronal cells, the DNA methylation assay also includes genomic DNA samples from glial cells that are present in both cultured and brain samples. Therefore, the fact that we still detect significant variations in CpG methylation further indicates that those changes represent drastic increases in DNA methylation that accompany reduced expression of Reelin in cortical neurons.

Prenatal restraint stress took place between days E11 and E20, which corresponds to 3.5–20 embryonic weeks in humans, and is assumed to be a timeframe when migration of neurons into the neocortex and hippocampal region occurs (with the exception of dentate granule cells where migration is accomplished later during development [54]). CR cells, the main source of Reelin in prenatal stages, are generated at E13 [54]. Concomitantly with cell migration, neuronal connectivity is developing during this period. Thus, the stressful maternal experience overlapped with the processes of lamination and establishment of basic connectivity of cortical regions in the offspring.

Reelin is produced principally in the embryonic cortex, amygdala, hippocampus and cerebellum [55]. Decreased cortical Reelin expression has been associated with depression, bipolar disorders and schizophrenia [30]. All of these pathological conditions display strong cortical dysfunctions, especially in the frontal cortex [56]. In addition, many studies evidence a strong connectivity between the amygdala, which is directly implicated in fear mechanisms and the response to stress [57], and the cerebral cortex [58]. Although the molecular pathways implicated in these disorders are still unknown, it has been found that in prenatal stressed rats, the inactive form of the kinase Gsk3-β decreases in prefrontal cortex, but not in hippocampus. The active form of Gsk3-β has been strongly related with psychiatric diseases [59]. This evidence suggests that only the cortex is altered by the pathogenic effects of this protein [16]. This is particularly interesting since the Reelin signaling pathway involves the modulation of Gsk3-β [20, 23]. Considering this evidence, we focused on some proteins downstream of the Reelin pathway, such as mDab1 and p35. mDab1 is one of the main Reelin effectors and its function has been related with cytoskeletal modulation and synaptogenesis [28, 60]. On the other hand, p35 is the main activator of Cdk5, a kinase involved in multiple functions during development of the nervous system [61, 62]. Furthermore, the kinase assay indicated a decrease of Cdk5 activity, that altogether with the Gsk3- β results reported by Szymanska et al. [16], suggests that PNS affects the expression of Reelin expression and its downstream components. It has been previously showed that Cdk5 phosphorylates C3G, a guanine-exchanging factor for the small GTPasebRap1b [63]. Interestingly, C3G participation in reelin-dependent neuronal migration is well documented [64]. As mentioned earlier, our *in vivo* results were replicated in our *in vitro* model, opening a promising avenue to study stress related mechanisms in cultured neurons.

In spite of decreased reelin-positive CR cells in stressed animals, cortical lamination appeared to be normal in PNS animals under microscopic visualization of Nissl stained samples (S1 Fig.). On the other hand, the reeler mouse, lacking Reelin, is characterized by a clear disorganization of the cortical layers [22]. According to the decrease in the Reelin expression observed under our experimental design, we propose that PNS induces a phenotype reminiscent of the heterozygous reeler mutants, although perhaps more dramatic, considering that the reduction of Reelin immunoreactivity may be higher than that observed in the heterozygote, which has a 50% of Reelin reduction in the anterior hemisphere [65]. Interestingly, the heterozygote displays milder neuroanatomical defects than those of Reelin null mutants [65] [66, 67], featuring reduced dendritic length and complexity [68, 69], and reduced dendritic spine density compared with wild type animals [70]. Additional alterations in postsynaptic structure in the reeler heterozygous, include decreases in scaffolding proteins, neurotransmitter receptors (NMDA receptor subunits NR2A and NR2B), and signaling proteins [71]. Furthermore, reelin deficiency is known to generate functional alterations. Reeler mice have impaired LTP and active-avoidance learning [72], while the reeler heterozygous has been described to have a deficit in LTP [73], as well as in a variety of behavioral tasks, including increased anxiety and motor impulsivity, and deficits in fear conditioning [74, 75], which are reminiscent of the findings reported here (but see below). Moreover, deficits in long-term potentiation (LTP) have been observed in preparations of animals deficient for the reelin receptors APOER2 or VLDRL [76]. There have been also experiments knocking down reelin signaling in the lateral entorhinal cortex, resulting in spatial memory deficits [77]. According to Folsom and Fatemi [74], the above impairments are also observed in autistic and schizophrenic patients. These findings are consistent with the idea that PNS generates a moderate disarrangement of cortical connectivity and plasticity that contributes to increased anxiety, and learning and memory impairments, which may be caused by a decrease in Reelin signaling.

In agreement with previous reports, we found that PNS-treated rats of both genders showed a significantly higher global activity compared to control animals during exploratory behavior tasks, like spontaneous locomotor activity and rearing [78–81]. This indicates that the timeframe used in this study was sufficient to produce persistent changes in offspring behavior [82–84]. In the elevated plus maze, PNS-treated animals showed more anxious behavior than control animals, with shorter periods of latency in the anxiogenic open arm [85, 86]. Animals from both groups evidenced similar number of entries to open and closed maze arms collectively, which excludes the possibility of a locomotor difference between groups. This evidence is consistent with previous reports showing that PNS is a generator of anxiogenic-like behaviors in the offspring when exposed to new places [12, 79, 87, 88]. We propose that our model imitates the reeler heterozygous, although there is no consensus with respect to the anxiogenic behavior in this mutant. Some authors have published no differences between the wild type and the heterozygous mice when animals are subjected to the open field and elevated plus maze test [47, 89]. Others have reported that heterozygous mice show lower levels of anxiety related behaviors in the elevated plus-maze compared to controls [90]. Additional reports indicate that heterozygous mice have increased levels of anxiety-related behaviors when performing the elevated plus maze [91]. Although the latter is consistent with our results, we cannot confirm if, and to what extent, the behavioral changes observed in our study can be attributed to a decrease in the Reelin protein.

The evaluation of learning and memory performance showed that both groups (control and PNS-treated rats) displayed conditioning in the passive avoidance test. However, contrary to controls, PNS-treated animals were unable to retain the conditioned response in days 4 and 5 post-shock, indicating a deficit in this kind of memory consolidation. This deficit is likely due to plasticity changes in the amygdala-prefrontal cortex circuit, which have been previously documented in stressed animals [92, 93]. Other studies have suggested a significant increase in anxious behavior, making it possible that the decreased latencies observed on days 4 and 5 post-shock (Fig. 8A) are due to an increased negative phototaxis that prompted them to search for the dark side of the box, rather than staying in the light side [94, 95]. However, this effect should have been evident in the recent memory tests as well, something that was not observed. Likewise, a reduction of contextual fear conditioned learning [91] and a deficit in reward-response learning have been demonstrated in reeler heterozygous mice [96]. Nevertheless, other studies also have indicated sex-specific responses [14, 97–99], which we were unable to detect. When comparing visual-spatial memory in the Morris Maze test between the two groups of animals, swimming performance revealed no differences. Likewise, one study has shown no significant differences between control and PNS treatments in the Morris maze test (Fig. 8b) [12], and similar results were obtained in reeler heterozygous mice [91, 96], indicating that PNS and Reelin signaling may not affect spatial memory. In contrast, other authors have observed impaired spatial learning in rat offspring after restraint prenatal stress [14, 100, 101]. A recent report indicates that PNS-treated mice do not display deficits in recent spatial memory in the Barnes maze test (1–4 days), which supports the present findings [102]. However, after 10 days these animals showed an increased persistence of this memory compared to controls, suggesting an extinction deficit, which was something that was not assessed in this study. Furthermore, remote memory performance was associated with a decrease in the firing rate of neurons within the medial prefrontal cortex, and with a disruption of functional connectivity in the hippocampal-prefrontal axis [102]. Although further studies must be performed to clarify the above discrepancies, it is tempting to postulate that they may be partly due to methodological differences, such as the strain of animals used, the period of pregnancy in which the protocol was implemented, the time of day the restriction was applied, the developmental stage when applying the paradigm, and the sensitivity of the test.

Many of the behavioral alterations observed in our studies involve emotional control and learning and memory mechanisms, which strongly depend on limbic structures like the amygdala and the hippocampus. Interestingly, most CR cells derive from the dorsal neuroepithelium and invade the hippocampus before reaching the neocortex. There are additional sources of CR cells, especially in the lateral hemisphere, in a region that overlaps with the developing claustro-amygdalar complex [103]. Thus, it will be interesting to focus further studies in the effects of PNS in Reelin expression, in regions such as the hippocampus and amygdala, in order to determine if Reelin mediates the observed behavioral changes.

While our results cannot establish causality, it is possible that a prenatal deficit in reelin signaling produces a long-term effect in the structure, function and plasticity of neural networks involved in learning and behavioral regulation that leads to the behavioral deficits we observed. This could be the consequence of (i) an enduring deficit in reelin expression in postnatal life that leads to network instability in the adult [19], and/or (ii) deficient neural-network development due to insufficient reelin signaling during critical perinatal periods, that would produce a cascade effect later in life. As Cajal-Retzius cells tend to disappear in postnatal development, reelin expression begins in GABAergic interneurons, in the neocortex and in the hippocampus. In the adult, reelin participates in the regulation of synaptic activity and plasticity by regulating NMDA receptors and calcium signaling. It will be of the foremost interest to determine whether the methylation effects observed in Cajal-Retzius cells of perinatal animals are also observed in the reelin-expressing interneurons in the adult, which will be addressed in future reports. Further studies, including analysis of the reeler heterozygote and the use of transgenic animals may also contribute to verify or disprove this causal link.

In summary, our work shows that PNS affects Reelin-positive neuron density in layer I (marginal zone) of all cortical areas, corresponding to CR neurons and elements of its signaling pathway. In agreement with previous reports, our results strongly indicate that this effect is at least partially induced, by CpG methylation at regulatory regions of the reelin promoter [104]. Also, we observe that post-natally, PNS-treated rats display anxious behavior, and learning and memory dysfunctions. Both impairments have a prenatal origin in which the neuronal gene expression and the correct establishment of neuronal connections were presumably affected, with subsequent behavioral consequences, in addition to possibly generating a predisposition for acquiring neuropsychiatric diseases. The relationship between PNS, the decrease of Reelin expression in CR neurons, and the anxious behavior accompanied with learning and memory impairments strongly place this study as a good model for investigating the origin and predisposition to acquired mental illnesses. Moreover, our *in vitro* model offers a new perspective in the investigation of specific molecular mechanisms that are involved in this process. Future studies will allow us to more precisely define the relationship between PNS, the decreasing number of Cajal-Retzius neurons in different brain structures, and behavioral impairments.

## Supporting Information

S1 Fig Comparable cortical cytoarchitectonic patterns in control and prenatally stressed rats using Nissl staining. MZ = marginal zone, UCP = upper cortical plate, LCP = lower cortical plate, L = cortical layer. Scale bar = 50 μm.
(TIF)
