# Supplementary figures and images for: Prenatal Stress Down-Regulates Reelin Expression by Methylation of Its Promoter and Induces Adult Behavioral Impairments in Rats
Source: PLoS One. 2015 Feb 13;10(2):e0117680. doi: 10.1371/journal.pone.0117680 (PMC4332679; doi:10.1371/journal.pone.0117680)

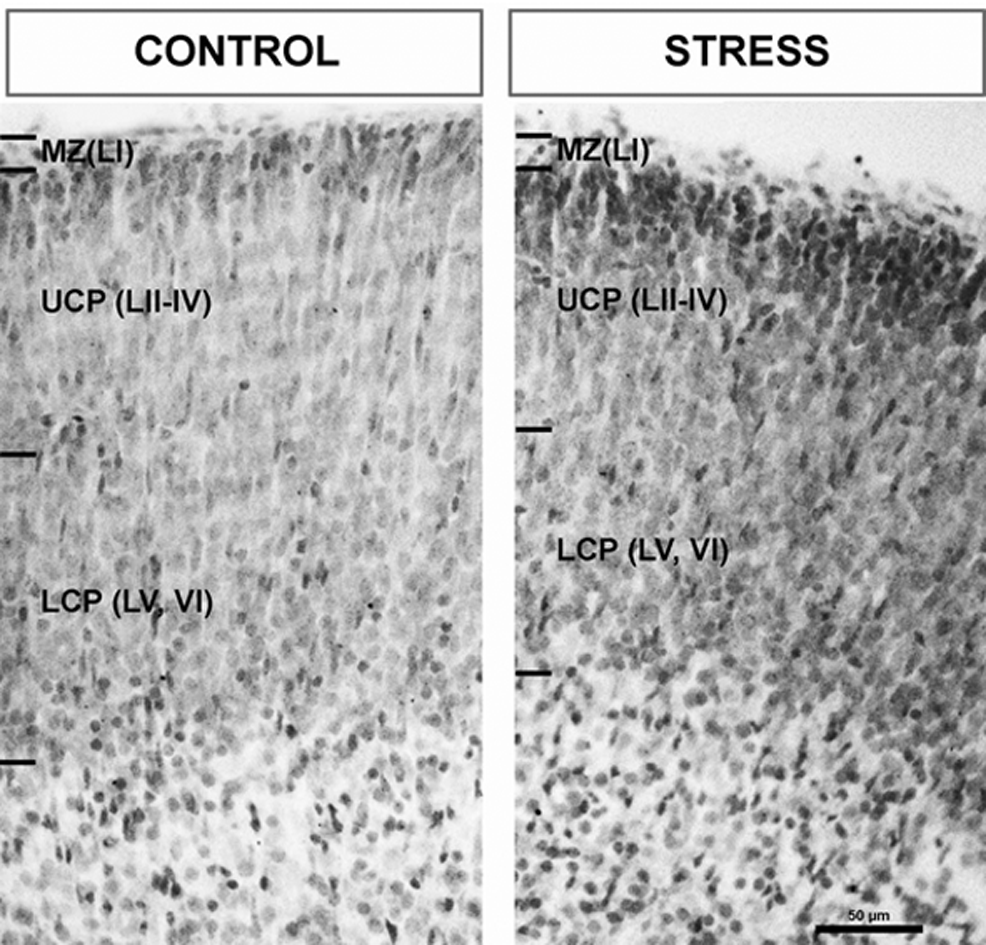

Supplement: S1 Fig — MZ = marginal zone, UCP = upper cortical plate, LCP = lower cortical plate, L = cortical layer. Scale bar = 50 μm. (TIF) [file pone.0117680.s001.tif]
